# Nutritional and productive parameters of Holstein/Zebu cows fed diets containing cactus pear

**DOI:** 10.5713/ajas.18.0584

**Published:** 2019-09-01

**Authors:** Lucas Daniel Alcântara Borges, Vicente Ribeiro Rocha Júnior, Flávio Pinto Monção, Camila Soares, José Reinaldo Mendes Ruas, Fredson Vieira e Silva, João Paulo Sampaio Rigueira, Natanael Mendes Costa, Laura Lúcia Santos Oliveira, Walber de Oliveira Rabelo

**Affiliations:** 1 Department of Animal Science and Technology, State University of Montes Claros, Avenue Reinaldo Viana, 2630, Janaúba, MG, 39440-000, Brazil; 2 National Institute of Science and Technology –INCT Animal Science Member, Brasília, 71605-001, Brazil

**Keywords:** Body Condition Score, Elephant Grass, Milk Production, Nitrogen Balance, Sorghum Silage

## Abstract

**Objective:**

This study ascertained effects of cactus pear in association with different roughage in the diet of F1 Holstein/Zebu cows on intake, nutrient digestibility, nitrogen balance, ingestive behavior and performance.

**Methods:**

Eight cows with 72±11 days of lactation were used. The experimental design was simultaneous in two 4×4 Latin squares. Four experimental diets were used: Diet 1, sorghum silage as exclusive roughage; Diet 2, sorghum silage associated with cactus pear in a proportion of 50% of the roughage (dry matter basis); Diet 3, elephant grass (*Pennisetum purpureum* cv. Roxo) as exclusive roughage; Diet 4, elephant grass associated with cactus pear in a proportion of 50% of the roughage. The roughage:concentrate ratio was 75:25.

**Results:**

Dry matter intake (p = 0.01) was higher with sorghum silage. There were differences in dry matter intake (p = 0.01), crude protein (p<0.01), ether extract (p = 0.01), non-fibrous carbohydrates (p<0.01) and total digestible nutrients (p = 0.01) among the diets. Cactus pear in the diet reduced water intake by 44.52% (p<0.01). The nitrogen balance was 59.71% and 27.49% lower in animals treated with exclusive sorghum silage and sorghum silage associated with cactus pear in relation to diets with elephant grass and elephant grass associated with cactus pear, respectively (p<0.01). The diets did not influence the milk production (p = 0.70), 3.5% fat corrected milk production (p = 0.72) or feed efficiency (p = 0.61).

**Conclusion:**

The association of cactus pear with sorghum or elephant grass silage does not alter milk production, reduces the intake of dry matter and water and improves the digestibility of nutrients.

## INTRODUCTION

Brazil has the largest commercial cattle herd in the world, with more than 218.2 million animals [1]. It is also the fifth largest milk producer (39.4 million tonnes), using crossbred Holstein/Zebu cows managed in a semi-intensive system with the use of tropical grasses in summer and supplemented with roughage and concentrate in the dry season [2]. In semiarid regions, mainly due to long periods of drought, animals are often managed with a diet supply in troughs, making food costs one of the highest costs of production [3]. This is due to the greater inclusion of concentrated ingredients (corn and soybean meal) in the diet, which costs more in relation to roughage forage [4].

As an alternative, cactus pear has been an important food resource in dairy production due to its adaptation to the edaphoclimatic conditions of the region, high water content, green mass production potential (241.75 t/ha green mass and 12.46 t/ha of dry matter [DM]) and nutritional value (as a source of energy, non-fibrous carbohydrates [NFCs]) [3–5] compared to traditional roughage sources. However, the exclusive use of cactus pear in diets for lactating cows is not recommended because of the low fiber content and crude protein (CP) [3–5], which can lead to metabolic disorders, low fat content in milk, low dry matter intake (DMI) and body weight (BW) loss [5]. Thus, Ben Salem et al [6] have suggested the use of cactus pear associated with other traditional roughage as silage, hay and fresh roughage in ruminant diets. However, there is a need to evaluate the changes in the nutritional, behavioral and productive parameters of lactating cows, especially in crossbred Holstein/Zebu cows, which have the genetic composition for milk production and are adapted to tropical climatic conditions [2], in addition to clarifying the efficiency of nutrient use.

The hypothesis is that the association of cactus pear with different types of roughage can improve dietary energy levels without modifying milk production in F1 Holstein/Zebu cows, despite a possible reduction in DMI. Therefore, the objective of this study was to evaluate the effect of sorghum silage or elephant grass cv. Roxo associated or not with cactus pear in diets of F1 Holstein/Zebu cows on lactation, nutrient intake and digestibility, nitrogen balance, productive performance and feeding behavior.

## MATERIALS AND METHODS

### Animal care and location

The experimental procedure was approved by the Institutional Committee on Animal Use of State University of Montes Claros (protocol number 138/2017).

### Site, period, facilities, and animals

The experiment was conducted at the State University of Montes Claros (Unimontes), Janaúba, Minas Gerais, Brazil (geographical coordinates: 15°52′38″ S, 43°20′05″ W). The experiment lasted 72 days, divided into four periods of 18 days, 14 days for the adaptation of the animals to the diets and management, and 4 for data collection and samples.

Animals were kept in individual pens (3×2 m) surrounded by a smooth wire with fiber-cement tile floors. The study included eight F1 Holstein/Zebu cows, with 72±11 days of lactation at the beginning of the experiment and a mean age of 72 mo. The experimental design was simultaneous in two 4×4 Latin squares, being four diets, four experimental periods and four animals.

### Experimental diets

Four experimental diets were used: Diet 1, sorghum silage as exclusive dietary roughage; Diet 2, sorghum silage associated with cactus pear in a proportion of 50% of the roughage (DM basis); Diet 3, elephant grass (*Pennisetum purpureum* cv. Roxo) as exclusive roughage; Diet 4, elephant grass associated with cactus pear in a proportion of 50% of the roughage (DM basis). The inclusion of cactus pear was based on the recommendation of Cavalcanti et al [7]. The diets were formulated to be isoprotein and to feed cows with an average of 550 kg of live weight and average production of 15 kg of milk corrected to 3.5% fat/d [8]. Urea was used to correct the CP contents of the diets [3] and a single concentrate was used in the four experimental diets. The roughage:concentrate ratio was 75:25. Feeding was performed twice a day at 8:00 am and 3:00 pm to allow for *ad libitum* intake, and the next feed was adjusted upward by 5% of the leftover every day from the DM provided.

The cactus pear ( *Opuntia ficus-indica* Mill, cv. Gigante) was harvested after 2 years of planting. The *Sorghum bicolor (L.)* Moench cultivar Volumax and the elephant grass were cultivated at the Unimontes Experimental Farm. The elephant grass was harvested when approximately 2.5 meters high.

### Chemical composition analysis

Samples of the supplied ingredients, leftovers and feces were analyzed for DM (method 967.03), crude ash (method 942.05), organic matter, CP (N×6.25) (method 988.05), and ether extract (EE; method 920.29 contents following the recommendations of the AOAC [9]. The contents of neutral detergent fiber corrected for ash and protein (NDFap; using heat-stable alpha-amylase without sodium sulfite) and acid detergent fiber (ADF) were determined as described by Van Soest et al [10], and the lignin content was determined by treating the ADF residue with 72% sulfuric acid. The NFC were estimated according to Detmann et al [11]. The contents of neutral detergent insoluble nitrogen and acid detergent insoluble nitrogen were estimated according to Licitra et al [12]. The total digestible nutrients (TDN) were estimated using the formula proposed by NRC [8].

The proportion of the ingredients and the chemical com position of the diets and the ingredients used during the experimental period can be verified in Table 1, 2.

### Nutrient intake, water, digestibility, and body condition score

The DMI was determined by the difference between the amounts of offered food and leftovers. During the experiment, every morning before offering the food, leftovers were collected and weighed, and the data were recorded for daily control.

Water intake was evaluated daily; the water was supplied as drinkable water with a capacity of 200 L. After 24 hours, the drinkers were completed, with the difference being considered as ingested by the animal. Two additional drinkers containing water were distributed near the animal cages in the shed and monitored to determine the daily evaporation. Total daily water intake was calculated as the sum of free water intake and presented as the diet minus evaporative loss and the leftovers in the drinking fountain.

To estimate the fecal DM production, indigestible ADF was used as an internal indicator. Fecal samples, food offered, and leftovers were sieved in a Willey mill with a 2-mm mesh sieve and packed in 7×7 cm non-woven fabric (NWF) bags (NWF–100 μ). The samples were placed in bags with 20 mg of DM/cm^2^ of surface and incubated in the rumen of fistulated cattle (different from initial group) for 288 h according to the recommendations of Detmann et al [11] (method INCT-CA F-008/1). The digestibility of nutrients, in percentage, was calculated using the following equation: (amount ingested – amount excreted in the feces)/amount ingested.

### Nitrogen balance

A single urine sample from each animal, named “*spot*”, was collected on the last day of each collection period approximately 4 h after the first feeding during spontaneous urination. A 10-mL urine sample was immediately diluted into 40 mL of sulfuric acid at 0.036 N, keeping the pH below 3 in order to avoid the decomposition of nitrogen compounds and the precipitation of uric acid. Samples from each animal were kept frozen at −20°C prior to the analysis.

The end-point method was used to estimate the creatinine concentration in urine via picrate and an acidifier (Doles commercial kits, Doles reagentes, Goiânia, GO, Brazil). To obtain daily creatinine [13] and urea [14] excretion, an average of 24.04 mg/kg of BW was used.

Samples of milk from each animal were collected twice daily for the last four days of each period and preserved with bronopol to quantify the levels of fat, milk protein, and milk urea nitrogen (MUN) by the infrared method. The concentrations of MUN were determined by enzymatic and spectrophotometric methods of transreflectance using a ChemSpeck 150 (Uniontown, OH, USA).

To calculate the nitrogen balance, the quantities of nitro gen ingested (g/d) and excreted in the feces, urine and milk were considered. The efficiency of dietary nitrogen utilization was calculated by dividing the concentration of nitrogen retained in milk by the nitrogen intake [15]. Feed efficiency was calculated by dividing the average milk production by the DMI [16].

### Evaluation of feeding behavior, performance and body condition score

The feeding behavior was assessed in the last 2 days of the trial period. For the evaluation of the feeding behavior, all animals were observed visually for 24 h, and the observations were recorded at 5-min intervals, which included eating, ruminating, and idle time [17]. On the same day, three observations were made for each animal: in the morning, at noontime, and at night. Data were collected by trained observers using digital timers. During the nocturnal observation, the environment was kept under artificial light. Feeding behavior variables (eating, ruminating, and idle time) were obtained by using equations adapted from Bürger et al [18].

The cows were mechanically milked twice a day at 7:00 am and 2:00 pm in the presence of the calf. During the last four days of each experimental period, the milk yields per cow were recorded. The 3.5% fat corrected milk production was calculated using the equation proposed by Sklan et al [19].

At the end of the experimental period, the animals were weighed without fasting from solid food to determine the final BW. Feed efficiency was calculated as the ratio between 3.5% fat corrected milk production (kg/d) and DMI (kg/d). Body condition score were evaluated by a single technician at the beginning and end of each experimental period using a scale of 1 to 5 points with intervals of 0.25 [20].

The evaluation of feeding costs was performed according to Rennó et al [21]. The costs per kg of DM of the diet ingredients were: sorghum silage, $0.057, cactus pear $0.028, elephant grass $0.042, and concentrate $0.46. The amounts were expressed in US dollars, considering the R $3.5 ratio for each $1.0.

### Statistical analysis

Data were evaluated by analysis of variance using the MIXED procedure of SAS [22], version 9.0 (SAS Inst. Inc., Cary, NC, USA). Data normality (Shapiro-Wilk test at 5% probability) was verified by the UNIVARIATE procedure in SAS. The statistical model used for analyses was Y_k(ij)_ = μ+P_i_+A_j_+T_k(ij)_ +PI+e_k(ij)_, where Y_k(ij)_ is the observation concerning the treatment “k”, within period i and animal j; μ is a constant associated with all observations; Pi is the effect of period i, with i = 1, 2, 3 and 4; A_j_ is the animal effect j, with j = 1, 2, 3, and 4; T_k(ij)_ is the treatment effect k, with k = 1, 2, 3 and 4; PI is the initial BW as covariable; and e_k(ij)_ is the experimental error associated with all observations (Y_k(ij)_), which is independent and by hypothesis has a normal distribution with mean zero and variance δ_2_. Treatments (T_k(ij)_) were considered as fixed effects; animals (A_j_), experimental period (P_i_), initial BW and the error term (e_k(ij)_) were random effects.

When significant by the F test, the means of treatments were compared by Tukey’s test. Mean values were considered different when p<0.05, and trend to the difference when 0.05 ≤p≤0.10.

## RESULTS

### Nutrients intake, water, and digestibility

There were differences in DMI (p = 0.01), CP (p<0.01), EE (p = 0.01), NFC (p<0.01), and TDNs (p = 0.01) among the diets (Table 3). The inclusion of cactus pear in the diet reduced water consumption by 44.52% in relation to the animal diets based on sorghum silage and elephant grass (p<0.01). The DM digestibility (p = 0.01) was 12.65% higher for sorghum silage+ cactus pear compared to the elephant grass base without cactus pear. The digestibility of CP (p = 0.01) was 11.85% higher in diets with the exclusive elephant grass base and associated with cactus pear in relation to the other diets (mean of 64.18%). The diet based on sorghum silage associated with cactus pear showed a higher TDNs value (p = 0.01) than the other diets.

### Nitrogen balance

Cows that received elephant grass in their diets presented nitrogen intakes 20.46%, 52.57%, and 31.91% higher than those of the animals fed diets based on sorghum silage, sorghum silage associated with cactus pear and elephant grass associated with cactus pear, respectively (p<0.01; Table 4). The nitrogen balance was 59.71% and 27.49% lower in animals treated with exclusive sorghum silage and sorghum silage associated with cactus pear in relation to diets with elephant grass and elephant grass associated with cactus pear, respectively (p<0.01). Cows fed sorghum silage associated with cactus pear showed better nitrogen use efficiency (p<0.01). However, animals that received diets based on elephant grass had a concentration of urea nitrogen in the milk 26.74% higher than those of the animals that received the other diets.

### Evaluation of feeding behavior, performance, and body condition score

Cows fed elephant grass only spent 54 minutes at feeding time (p = 0.01) compared to the other animals of the other treatments (Table 5). The animals fed with cactus pear, regardless of the associated roughage, presented less spent time for rumination (6.53 hours) and, consequently, 2.91 h/d more idle in relation to the other animals. The feed efficiency of DM (p<0.01), in g/h, was higher in diets with sorghum silage, while the feed efficiency of the NDFap (p<0.01) was lower with the cactus pear-containing diets.

The diets did not influence milk production (p = 0.70), 3.5% fat corrected milk production (p = 0.72), final weight (p = 0.60), feed efficiency (p = 0.61) or DM content (0.79 kg of milk/kg of dairy cows), with means of 12.57 kg/d, 14.30 kg/d, 549.75 kg, 1.21 kg of DM/kg of milk and 0.79 kg of DM, respectively (Table 6). In the BW differential (p<0.01), it was found that cows fed diets containing sorghum silage gained 2.91% by weight relative to the initial BW. The body score differential also varied (p<0.01) among the diets. The inclusion of cactus pear in the diet reduced 20.25% and 5.72% when compared to diets with sorghum silage and elephant grass as source of roughage, respectively.

## DISCUSSION

The isolated action or interaction between the physical (ruminal filler), physiological and psychogenic factors in an animal interferes with the DMI [4]. Therefore, the higher DMI in cows receiving sorghum silage as an exclusive source of fiber is justified by the higher DM content of the diet in relation to the others. In general, diet DMI was higher than the 13.75 kg/d recommended by the NRC [8]. Murta et al [23] also observed DMI in crossbred Holstein/Zebu cows in lactation was higher than that recommended by NRC [8] and similar to that of this study. It was verified that with the inclusion of the cactus pear in the diet, the intake of NDFap by the animals decreased slightly, and the small variation was not sufficient to interfere with the rumination processes of the animals. This occurred because the NDF content of cactus pear (300 g/kg of DM) is lower than that of sorghum silage and elephant grass (above 600 g/kg DM). Mertens [24] recommended an intake of 6.6 kg/d of NDF to guarantee the process of rumination and to avoid metabolic disturbances in the animals. Thus, even with the inclusion of cactus pear in the diets of cows, the intake of NDFap met the recommendations.

The presence of cactus pear in the diet improved nutrient digestibility substantially, which is related to the high content of NFCs that, when associated with other carbohydrates such as the starch present in sorghum silage, provides a greater amount of energy for the fibrolytic bacteria to degrade the potentially digestible DM [5]. Moreover, the presence of ruminal ammonia and carbon skeletons are also used for the synthesis of microbial protein, which is essential in the nutrition of ruminants as the main source of amino acids and peptides [16]. This higher ruminal ammonia production may have occurred in the diets with exclusive elephant grass or associated with cactus pear due to the greater digestibility of the CP, which in turn may be related to the higher intake and composition (fraction A) of this nutrient and, possibly, to the greater degradability of the protein of this forage in relation to sorghum silage [5]. Another relevant aspect observed in this study was the lower daily intake of water in the animals that received diets containing cactus pear, mainly in regard to semiarid regions, where water restrictions are a reality and limit animal production.

As for the nitrogen balance, the higher value of nitrogen retained with the elephant grass diet as exclusive roughage is justified by the higher intake of nitrogen in the diet; however, the efficiency of dietary nitrogen utilization was lower, and the MUN was higher. These results suggest that the inclusion of cactus pear as a source of NFC increased the availability of energy for microbial synthesis and consequently improved the utilization of dietary nitrogen. According to Doska et al [25], the mean value of MUN in a dairy herd should be between 10 and 14 mg/dL; values above 14 mg/dL would indicate deficiency in the fermentation of NFCs, excess protein in the diet and/or imbalance between the availability of energy and nitrogen. Only the elephant grass diet presented a value above the limit (16.07 mg/dL), which is explained by the lower intake of nonfibrous carbohydrates and the higher intake of CP. The longer feeding time in the elephant grass diet is justified by the higher fiber content and larger particle size of the grass. The lowest rumination time with cactus pear inclusion is due to the high content of NFCs of high fermentation, thus increasing the rate of passage, decreasing the rumination time, and directly influencing the time spent idle. This change in behavior may favor the productive efficiency of the animal, with the decrease in maintenance requirements, especially when the ambient temperature is high [23]. The lower DM and NDF contents of the diets containing cactus pear, in addition to the higher digestibility of the diets in relation to the others evaluated, influenced the rumination characteristics of the cows, thus reducing the number of rumined bolus and the number of chews per bolus. The higher DM feed efficiency, in g/h, with diets containing silage is explained by the higher DMI, as well as the higher DM content of sorghum silage. The feed efficiency of NDF, in g/h, was lower in the diets with cactus pear because of the lower NDF contents in these diets. On the other hand, the inclusion of cactus pear favored the rumination efficiency of DM due to the greater proportional reduction of rumination time. The higher rumination efficiency of NDF with grass-based diets is mainly due to the higher NDF content, as well as the lower digestibility and physical characteristics of this forage.

Despite differences in the consumption and digestibility of the diets with or without cactus pear, milk production, milk production corrected to 3.5% fat and feed efficiency were similar among the diets. Therefore, the diet based on elephant grass associated with cactus pear presents lower food costs, which may imply an increase in profitability.

## CONCLUSION

The association of cactus pear with sorghum silage or elephant grass, *in natura*, did not modify milk production, reduce nutrients intake and water and improve digestibility on F1 Holstein/Zebu cows.

## Figures and Tables

**Table 1:** Proportion of the ingredients of the experimental diets and chemical composition of the diets

Item	Experimental diets			

	Sorghum silage	Elephant grass	Sorghum silage+cactus pear	Elephant grass+cactus pear
Proportion of ingredients in diets (g/kg DM)				
Sorghum silage	750.00	0.00	375.00	0.00
Elephant grass	0.00	750.00	0.00	375.00
Cactus pear	0.00	0.00	375.00	375.00
Corn ground grain	170.10	170.10	170.10	170.10
Soybean meal	71.20	71.20	71.20	71.20
Mineral mix1)	8.70	8.70	8.70	8.70
Chemical composition (g/kg DM)				
Dry matter	474.10	381.90	380.70	334.60
Crude protein2)	111.70	114.90	111.80	112.20
Ether extract	24.40	25.30	22.70	22.50
Non-fibrous carbohydrates	253.50	200.90	406.8	391.70
NDFap	523.90	568.90	381.20	404.30
Lignin	83.20	84.30	64.40	65.00

DM, dry matter; NDFap, neutral detergent fiber corrected for ash and protein.

1) Mineral Mix, content per kg of product: calcium (128 g), phosphorus (100 g), sodium (120 g), magnesium (15 g), sulfur (33 g), cobalt (135 mg), iron (938 mg), iodine (160 mg), manganeses (1,800 mg), selenium (34 mg), zinc (5,760 mg), fluorine (1,000 mg).

2) Mean concentrations of urea/ammonium sulfate (9:1) in the dry matter of the roughage fractions of the diets: 7.20 g/kg (sorghum silage), 10.70 g/kg (sorghum silage associated with cactus pear), 3.00 g/kg (elephant grass), 6.00 g/kg (elephant grass associated with cactus pear).

**Table 2:** Chemical composition of the ingredients used in the formulation of the diets

Item (g/kg DM)	Elephant grass	Sorghum silage	Cactus pear	Corn ground grain	Soybean meal
Dry matter	200.9	332.0	83.0	901.5	900.1
Crude ashes	100.0	78.0	44.0	21.0	60.0
Organic matter	900.0	922.0	954.0	979.0	940.0
Crude protein	80.30	65.5	48.0	92.0	469.0
Ether Extract	15.0	15.5	11.0	43.0	58.8
Non-fibrous carbohydrates	75.8	213.6	610.0	644.0	254.2
Neutral detergent fiber	714.0	650.2	300.1	204.0	161.7
NDFap	699.0	639.0	287.0	200.0	158.0
Acid detergent fiber	462.0	389.0	190.2	59.0	112.2
Lignin	103.5	100.2	52.0	29.0	25.3

DM, dry matter; NDFap, neutral detergent fiber corrected for ash and protein.

**Table 3:** Intake, digestibility of nutrients and water consumption for F1 Holstein/Zebu cows fed different diets containing or not cactus pear

Item	Diets1)				SEM	p-value

	Sorghum silage	Elephant grass	Sorghum silage+ cactus pear	Elephant grass+ cactus pear		
Intake						
Dry matter (kg/d)	19.4^a^	15.84^b^	15.91^b^	14.91^b^	0.85	0.01
Crude protein (kg/d)	1.77^b^	2.13^a^	1.40^c^	1.62^bc^	0.09	<0.01
Ether extract (kg/d)	0.40^a^	0.33^ab^	0.30^b^	0.29^b^	0.02	0.02
NFC (kg/d)	5.51^b^	2.88^c^	6.84^a^	5.71^ab^	0.29	<0.01
NDFap (kg/d)	9.94^a^	8.96^a^	6.27^b^	6.17^b^	0.43	<0.01
TDN (kg/d)	9.98^a^	7.66^b^	9.67^ab^	8.34^ab^	0.52	0.01
Dry matter (% BW)	3.44^a^	2.86^ab^	2.92^ab^	2.78^b^	0.15	0.02
Crude protein (% BW)	0.32^b^	0.39^a^	0.26^b^	0.30^b^	0.02	<0.01
NFC (% BW)	0.977^b^	0.52^c^	1.26^a^	1.06^b^	0.05	<0.01
NDFap (% BW)	1.76^a^	1.62^a^	1.15^b^	1.15^b^	0.08	<0.01
TDN (% BW)	1.79^a^	1.38^b^	1.78^a^	1.55^ab^	0.08	<0.01
Water (L/d)	49.30^a^	48.77^a^	31.27^b^	23.14^b^	3.98	<0.01
Digestibility (%)						
Dry matter	62.76^ab^	55.74^b^	68.39^a^	58.66^ab^	2.50	0.01
Crude protein	60.01^b^	73.26^a^	68.36^ab^	70.33^a^	2.45	0.01
Ether extract	15.34^b^	15.97^b^	30.39^a^	19.56^ab^	3.35	0.02
NFC	62.26^ab^	59.61^b^	73.10^a^	72.79^a^	4.07	0.05
NDFap	55.84^ab^	48.75^b^	57.61^a^	49.48^ab^	2.56	0.05
TDN	52.41^ab^	49.11^b^	61.19^a^	56.45^ab^	2.36	0.01

SEM, standard error of the mean; p, probability; NFC, non-fibrous carbohydrates; NDFap, neutral detergent fiber corrected for ash and protein; TDN, total digestible nutrients; BW, body weight; DM, dry matter.

1) Sorghum silage; Sorghum silage associated with cactus pear (*Opuntia fícus indica* cv. Gigante, 50% of DM); Elephant grass *in natura* (*Pennisetum purpureum* cv. Roxo); Elephant grass associated with cactus pear.

a–c Means followed by equal letters do not differ by Tukey’s test (p<0.05).

**Table 4:** Balance and efficiency of nitrogen utilization in F1 Holstein/Zebu cows fed different diets containing or not cactus pear

Item	Diets1)				SEM	p-value

	Sorghum silage	Elephant grass	Sorghum silage+ cactus pear	Elephant grass+ cactus pear		
N-ingested (g/d)	283.92^b^	342.01^a^	224.17^d^	259.27^c^	4.64	<0.01
N-milk (g/d)	80.99^a^	69.47^a^	77.15^a^	72.11^a^	5.47	0.46
N-feces (g/d)	104.78^a^	91.41^ab^	71.67^b^	77.87^ab^	8.37	0.05
N-urine (g/d)	21.95^a^	10.92^a^	14.40^a^	14.71^a^	4.41	0.37
Nitrogen balance (g/d)	76.20^c^	170.21^a^	60.95^c^	94.58^b^	103	<0.01
NUE	0.29^b^	0.21^c^	0.35^a^	0.28^b^	0.02	<0.01
UUN (mg/dL)	21.89^a^	21.27^a^	23.38^a^	20.77^a^	0.76	0.11
MUN (mg/dL)	12.45^b^	16.07^a^	10.97^b^	11.90^b^	0.81	<0.01

SEM, standard error of the mean; p, probability; NUE, nitrogen use efficiency; UUN, urinary urea nitrogen; MUN, milk urea nitrogen.

1) Sorghum silage; Sorghum silage associated with cactus pear (*Opuntia fícus* indica cv. Gigante, 50% of dry matter); Elephant grass *in natura* (*Pennisetum purpureum* cv. Roxo of Botucatu); Elephant grass associated with cactus pear.

a–c Means followed by equal letters do not differ by Tukey’s test (p<0.05).

**Table 5:** Ingestive behavior of F1 Holstein/Zebu cows fed different diets containing or not cactus pear

Item	Diets1)				SEM	p-value

	Sorghum silage	Elephant grass	Sorghum silage+ cactus pear	Elephant grass+ cactus pear		
Feeding						
h/d	5.27^b^	6.14^a^	5.00^b^	5.41^ab^	0.22	0.01
min/kg DM	16.75^c^	24.54^a^	19.70^bc^	22.35^ab^	0.98	<0.01
min/kg NDFap	32.79^b^	43.59^ab^	54.01^a^	54.03^a^	3.93	<0.01
Rumination						
h/d	9.21^a^	8.68^a^	6.45^b^	6.62^b^	0.27	<0.01
min/kg DM	28.55^b^	34.55^a^	23.96^c^	27.47^bc^	1.03	<0.01
min/kg NDFap	55.77^a^	61.43^a^	65.46^a^	66.40^a^	4.14	0.28
Chewing						
number/bolus	62.90^a^	54.09^ab^	52.45^ab^	51.23^b^	2.74	0.03
number/d	33,962^a^	31,224^a^	22,620^b^	23,829^b^	738	<0.01
h/d	14.48^a^	14.83^a^	11.45^b^	12.04^b^	0.36	<0.01
min/kg DM	45.30^bc^	59.09^a^	43.67^c^	49.82^b^	1.33	<0.01
min/kg NDFap	88.57^b^	105.03^ab^	119.47^a^	120.44^a^	7.18	0.02
Idleness						
h/d	9.51^b^	9.16^b^	12.54^a^	11.95^a^	0.36	<0.01
Feed efficiency						
g DM/h	3,748^a^	2,592^b^	3,297^a^	2,762^b^	133	<0.01
g NDFap/h	12,128^a^	11,421^a^	8,104^b^	9,807^b^	646	<0.01
Rumination efficiency						
g DM/h	2,115^b^	1,819^c^	2,531^a^	2,269^b^	58	<0.01
g NDFap/h	7,007^ab^	7,997^a^	6,384^b^	8,000^a^	277	<0.01

SEM, standard error of the mean; p, probability; DM, dry matter; NDFap, neutral detergent fiber corrected for ash and protein.

1) Sorghum silage; Sorghum silage associated with cactus pear (*Opuntia fícus indica* cv. Gigante, 50% of the dry matter); Elephant grass *in natura* (*Pennisetum purpureum* cv. Roxo of Botucatu); Elephant grass associated with cactus pear.

a–c Means followed by equal letters do not differ by Tukey’s test (p<0.05).

**Table 6:** Productive performance and feed efficiency of F1 Holstein/Zebu cows fed different diets containing or not cactus pear

Item	Diets1)				SEM	p-value

	Sorghum silage	Elephant grass	Sorghum silage+ cactus pear	Elephant grass+ cactus pear		
Milk production (kg/d)	13.62	12.09	12.27	12.33	1.02	0.70
3.5% fat corrected milk production (kg/d)	15.31	13.47	14.48	13.97	1.17	0.72
Final body weight (kg)	565	556	544	534	16.88	0.60
Body weight change (kg)	16.18^a^	−6.87^b^	7.81^ab^	−18.75^b^	5.86	<0.01
Initial body condition score	2.81^b^	3.06^a^	2.56^ab^	2.81^ab^	0.12	0.06
Final body condition score	3.1^a^	2.8^ab^	2.7^ab^	2.6^b^	0.10	0.01
Body condition score change	0.3^a^	−0.2^b^	0.1^ab^	−0.2^b^	0.10	<0.01
Feed efficiency (kg milk/kg DM)	0.72	0.78	0.81	0.86	0.07	0.61
Food cost ($/d)	3.85	2.97	3.07	2.80	0.31	-

SEM, standard error of the mean; p, probability; DM, dry matter.

1) Sorghum silage; Sorghum silage associated with Cactus pear (*Opuntia fícus indica* cv. Gigante, 50% of DM); Elephant grass *in natura* (*Pennisetum purpureum* cv. Roxo of Botucatu); Elephant grass associated with cactus pear

a,b Means followed by equal letters do not differ by Tukey’s test (p<0.05).
